# Glucose Homeostasis, Fetal Growth and Gestational Diabetes Mellitus in Pregnancy after Bariatric Surgery: A Scoping Review

**DOI:** 10.3390/jcm9092732

**Published:** 2020-08-24

**Authors:** Ellen Deleus, Bart Van der Schueren, Roland Devlieger, Matthias Lannoo, Katrien Benhalima

**Affiliations:** 1 Department of Abdominal Surgery, University Hospital Gasthuisberg, KU Leuven, Herestraat 49, 3000 Leuven, Belgium; ellen.deleus@uzleuven.be (E.D.); matthias.lannoo@uzleuven.be (M.L.); 2 Department of Endocrinology, University Hospital Gasthuisberg, KU Leuven, Herestraat 49, 3000 Leuven, Belgium; bart.vanderschueren@uzleuven.be; 3 Department of Chronic Diseases, Metabolism and Ageing, KU Leuven, Herestraat, 49, 3000 Leuven, Belgium; 4 Department of Obstetrics & Gynaecology, University hospital Gasthuisberg, KU Leuven, Herestraat 49, 3000 Leuven, Belgium; roland.devlieger@uzleuven.be

**Keywords:** bariatric surgery, pregnancy outcome, glucose homeostasis, gestational diabetes mellitus, gastric bypass, sleeve gastrectomy, small-for-gestational age, self-monitoring of blood glucose, continuous glucose monitoring

## Abstract

Background: Pregnancies in women with a history of bariatric surgery are becoming increasingly prevalent. Surgically induced metabolic changes benefit mother and child, but can also lead to some adverse pregnancy outcomes. Knowledge about glucose homeostasis in these pregnancies could elucidate some of the mechanisms behind these outcomes. This review focusses on glucose homeostasis and birth weight. Methods: We considered papers dealing with glucose homeostasis, gestational diabetes mellitus (GDM) and/or small-for-gestational age infants (SGA) in pregnancies with a history of sleeve gastrectomy (SG) or Roux-en-y gastric bypass (RYGB). Results: Since an OGTT is unreliable to diagnose GDM in a pregnancy after bariatric surgery, the true incidence of GDM is unknown. Alternative screening strategies are needed. Furthermore, these pregnancies are marked by frequent hypoglycemic events as well as wide and rapid glycemic excursions, an issue that is very likely underreported. There is a lack of uniformity in reporting key outcomes and a large variation in study design and control population. Conclusion: Alteration of glucose homeostasis in a pregnancy after bariatric surgery should be further studied using unequivocal definition of key concepts. Glycemic control may prove to be a modifiable risk factor for adverse pregnancy outcomes such as the delivery of an SGA baby.

## 1. Introduction

The prevalence of obesity continues to rise worldwide: in 2015 it was estimated to be 5% among children and 12% among adults. In every age group, women are more affected than men [1]. In a Belgian survey from 2018, 15.9% of the general population had obesity (body mass index (BMI) ≥ 30 kg/m^2^), in comparison to 10.8% in 1997 [2]. Maternal obesity is also rising, with the Euro-Peristat (perinatal health information in Europe) survey indicating a median prevalence of maternal obesity of 13.2% [3]. Maternal obesity during pregnancy is associated with increased risk of miscarriage, congenital anomalies, gestational diabetes mellitus (GDM), macrosomia, caesarian section, hypertensive disorders and admission to neonatal intensive care unit (NICU) [4,5]. For women with a BMI of ≥ 40 kg/m² or a BMI of ≥35 kg/m² with comorbidities, bariatric surgery is the most effective long-term treatment [6]. The International Federation for Surgery of Obesity and Metabolic Disorders (IFSO) global registry report showed that between the period 2014 and 2018, 73.7% of all patients undergoing bariatric surgery was female. Median age at the time of operation was 42 years [7]. This confirms other reports that show that more than 50% of bariatric surgeries are performed in women of childbearing age [8].

The most commonly performed bariatric surgeries are currently Roux-en-Y gastric bypass (RYGB) and sleeve gastrectomy (SG). Laparoscopic adjustable gastric banding (LAGB) has been largely abandoned because of high long-term failure and removal rate [9,10]. Bariatric surgery has historically been divided into malabsorptive and restrictive procedures. However, based on vast scientific evidence from both animal and human research, this labeling does not reflect the mechanisms of action [11]. Both RYGB and SG are associated with distinct glycemic patterns marked by postprandial hyperinsulinemic hypoglycemia, nightly hypoglycemia and wide glycemic variability [12,13]. Improvement in insulin sensitivity as marked by improvement in HOMA-IR index (homeostatic model assessment for insulin resistance index) contributes largely to an improved glucose homeostasis [14]. On the other hand, β-cell function does not seem to recover after RYGB [15]. The Swedish Obese Subjects (SOS) trial showed a 75% decrease in new onset type 2 diabetes mellitus 2 and 10 years after bariatric surgery [16]. Moreover, the improvement in glucose homeostasis occurs before significant weight loss. A prospective randomized trial from Switzerland in non-pregnant patients showed that HOMA-IR index was significantly reduced a week after surgery. The effect at one week postoperative was more pronounced after RYGB, however after three months most SG and RYGB subjects had similar insulin resistance to lean controls [17]. This improvement has been attributed to an improved glucose-mediated incretin release [18]. However, this comes with a price, since both RYGB and SG are marked by enhanced glycemic variability and frequent occurrence of hypoglycemia [13,19,20].

Pregnancy after maternal bariatric surgery is a specific entity; it holds benefits as well as possible harms for both mother and child. Four recent meta-analyses examined the specific risks and benefits of a pregnancy after bariatric surgery (Table 1). Pregnancies after bariatric surgery, as compared with non-surgical controls, were associated with lower risk of GDM (OR 0.20–0.47) [21,22,23]; lower risk of macrosomia/large-for-gestational age (LGA) infants (OR 0.31–0.46) [8,21,22,23] and lower risk of hypertensive disorders of pregnancy (HDP) (OR 0.38–0.45) [21,22,23]. However other outcomes were worse; all four meta-analyses reported a higher risk for small-for-gestational age (SGA) (OR 1.93–2.23) [8,21,22,23]. Three meta-analyses also reported a higher risk for preterm birth (PB) (OR 1,31–1,35) [8,21,22], and two reported a higher risk for perinatal death (PD) (OR 1.05–1.38) [8,21].

In pregnancy, the effect of low glucose levels on unfavorable pregnancy outcomes are poorly studied. A different intrauterine environment may be responsible for an infant to be SGA [24,25]. In the 1970s and 1980s, several authors report on the association between maternal hypoglycemia during oral glucose tolerance test (OGTT) and intra uterine growth restriction [26,27,28]. A recent paper in a non-bariatric surgery population confirmed a significant association between low fasting plasma glucose and hypoglycemia during standard 2 h 75 gr OGTT and low birth weight [29].

Consequently, changes in glucose homeostasis after bariatric surgery may play an important role in observed outcomes. In a normal pregnancy, insulin sensitivity shifts during gestation. Early gestation is marked by an increased insulin sensitivity. When gestation progresses, a combination of maternal and placental hormones induce a state of insulin resistance [30]. As such, the fetus receives more glucose which, in turn, drives fetal production of insulin, an important growth factor in fetal live [31]. Since bariatric surgery improves insulin resistance, this may explain the improvement in GDM diagnosis. However, it could also be linked to the increased risk for SGA, since the level of insulin resistance in the later stages of gestation could be insufficient to provide enough glucose flux to the fetus.

About 80% of GDM cases in normal pregnancy are related to β-cell dysfunction on a background of chronic insulin resistance. The rate of GDM in the general population worldwide is about 16.5% [30]. However, there is a great variation in the diagnostic criteria for GDM worldwide [32,33]. In 2010, the ‘International Association of Diabetes and Pregnancy Study Groups’ (IADPSG) published recommendations for GDM screening during pregnancy. A 2 h 75 g OGTT between 24 and 28 weeks is recommended.

The purpose of this scoping review is to explore the existing evidence on the specific changes in glucose homeostasis as a result of the combined impact of pregnancy and altered gastro-intestinal physiology after RYGB and SG. In addition, data on the influence of altered glucose handling on fetal growth and diagnosis of GDM are analyzed.

## 2. Methods

### 2.1. Data Search

We applied the PRISMA guidelines for Scoping Reviews [34]. Between 15th January 2020 and 17th February 2020, a PubMed search was performed. The search was limited to research on humans, published after 2010. Studies on both singleton and multiple pregnancies were included. Since RYGB and SG are the most commonly performed bariatric surgery procedures, these search terms were used. However, data on other types of bariatric surgery were also included since it was often impossible to extract data specifically for SG and RYGB. The following search terms were used: (“Pregnancy”(Mesh) AND “Glucose”(Mesh) AND “Bariatric Surgery”(Mesh) OR “Gastric Bypass”(Mesh) OR “Gastrectomy”(Mesh) AND “Hypoglycemia”(Mesh) OR “Glucose”(Mesh)); (“Infant, Small for Gestational Age”(Mesh) AND “Bariatric Surgery (Mesh) OR “Gastric Bypass”(Mesh) OR “Gastrectomy”(Mesh) AND “Nutrient”(Mesh)); (“Bariatric Surgery”(Mesh) OR “Gastric Bypass”(Mesh) OR “Gastrectomy”(Mesh) AND “Diabetes, Gestational”(Mesh)). In addition, we hand-searched the reference lists of all selected articles and reviews. Exclusion criteria included no full text, full text not in English or Dutch, opinion, editorial, case report, not relevant or animal studies. Duplicates were removed. Certain clinical practice guidelines and narrative reviews were only used to underline arguments and were therefore not mentioned in the flow chart of included articles.

### 2.2. Data Analysis

We included original and review articles. Since this is not a systematic review, no quality analysis of the selected studies was done. We did not use a review protocol, data charting was done independently. We performed a descriptive analysis of all original articles using the following data: year, type of study, size of patient group, type of bariatric surgery performed, type of control group and outcome variables. Numbers reported in tables represent total number of pregnancies after bariatric surgery. Glycemic values are reported in mg/dL.

All four systematic reviews with meta-analysis are summarized in Table 1 with odds ratios regarding: macrosomia/LGA, GDM, hypertensive disorders of pregnancy, SGA, preterm birth and perinatal death.

## 3. Literature Search and Overview of Selection

**Figure 1:**
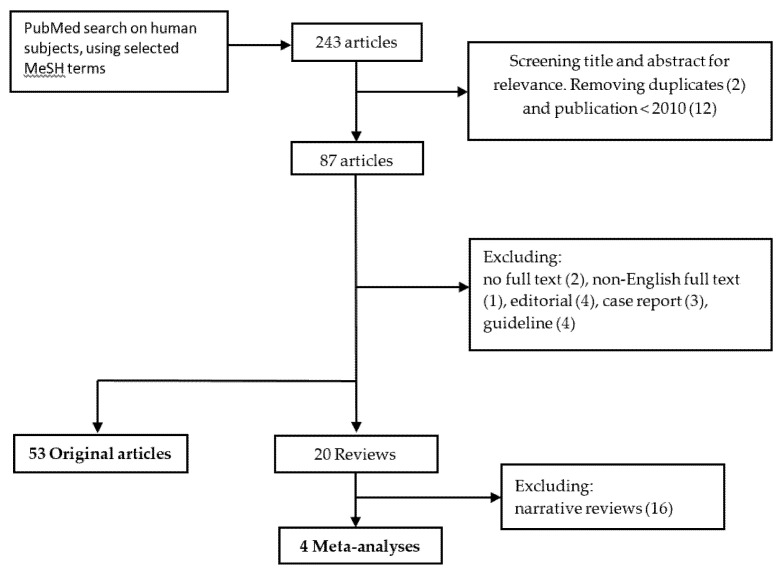
Literature search and selection process.

Initially, a total of 243 articles was found. After screening of titles and abstracts on relevance for the topic, we included 101 articles. We excluded 14 articles because of duplicate or publication before 2010. After applying exclusion criteria we retained 57 papers: 53 original articles and four meta-analyses (Figure 1).

## 4. Results

### 4.1. Characteristics of Glucose Homeostasis in Pregnancy After Bariatric Surgery

We identified nine observational studies that report on glycemic levels during a pregnancy after bariatric surgery (Table 2). Seven studies had a study population of less than 50 participants.

The most common technique to evaluate maternal glucose homeostasis in a pregnancy after bariatric surgery was a 75 or 100 g OGTT [35,37,38,39,40,41,42,43]. Consistently, a high prevalence of hypoglycemic events during the OGTT was reported, ranging from 5.26% to 90% of all patients. A retrospective cohort study from 2016 described glucose levels during an OGTT in pregnant RYGB women and BMI-matched, lean and obese controls. Mean fasting glucose was significantly lower in pregnant women after RYGB (74.95 mg/dL). Pregnant women after RYGB had a glycemic rise at 60 min, followed by hypoglycemia (<60 mg/dL) at 120 min, occurring in 54.8% of cases. When considering glycemic levels at 120 min (and not at 60 min) only 1.6% met the IADPSG criteria for GDM. When considering glycemic levels at 60 min, 43.5% of the post-RYGB women met the IADPSG criteria for GDM, however 39.3% of these women developed hypoglycemia at 120 min. The control group of obese women had no hypoglycemic episodes [37]. The highest rates of hypoglycemic events were reported in a population of RYGB patients that underwent an extended and frequently sampled 3 h 75 g OGTT after at least 8 h of fasting. Ninety percent of these women developed hypoglycemia (<50 mg/dL). The mean plasma glucose nadir in the RYGB group was 42.5 mg/dL [39]. After a 3 h 100 g OGTT, another group confirmed that the nadir was reached in 2 h in 42.4% and only after 3 h in 57.6%. Hypoglycemia was most commonly seen after RYGB (83.3%) and SG (54.5%), and less after LAGB (11.8%) [43].

A prospective observational study from the UK compared maternal insulin resistance in pregnant women after bariatric surgery versus pregnant women with similar BMI. A reduced insulin resistance, as assessed by HOMA-IR index, was found in pregnancies after bariatric surgery. The authors conclude that the positive effect of bariatric surgery on insulin resistance cannot solely be explained by weight reduction [41].

Two studies performed an intravenous glucose tolerance test (IVGTT) [39,40]. In 2017, Göbl et al. showed that IVGTT-derived insulin response was comparable between post RYGB pregnant women and normal weight pregnant women. They concluded that reactive hypoglycemia noticed after OGTT must be attributed to the specific anatomical alterations after gastric bypass surgery. An IVGTT reflects only the effect of plasma glucose on insulin release by the β-cell, whereas an OGTT reflects the additional effect of the altered gastro-intestinal tract on insulin release [39]. Leutner et al. also described an exaggerated expression of GLP-1 just before the occurrence of a hypoglycemic event in pregnant women after RYGB. This suggests that GLP-1 might be the main driver of this postprandial hyperinsulinemic hypoglycemia [40].

There are only two observational studies available with data on continuous glucose monitoring (CGM) during pregnancy after bariatric surgery [36,40]. In addition, there is one case report on the subject [44]. All three studies reported on CGM during pregnancy after RYGB. Bonis et al. described CGM in 35 RYGB pregnant women at 26.2 ± 5 weeks and reported wide and rapid changes in postprandial interstitial glucose (IG), as well as frequent hypoglycemia. The authors compared there results with CGM data from other studies in non-operated pregnant women and found these profiles to be very different. They showed that pregnant women after bariatric surgery have a lower mean fasting IG, similar 1 h postprandial IG and significantly lower 2 h postprandial IG. The postprandial IG peak occurred earlier, and the value was higher compared to non-operated women. The authors suggest therefore that a 75 g OGTT is probably a poor diagnostic tool for GDM, since baseline and 2 h value will be lower, and 1 h value will not be representative of the highest value [36]. In a study by Leutner et al., it was shown that pregnant women with previous RYGB had both the highest and the lowest mean IG values when comparing with non-operated normal-weight and obese pregnant women on a week-long CGM. The CGM (iPro2, Medtronic MiniMed, Northridge, California, USA) was blinded to the participants and only retrospectively analyzed by the investigators. Glucose profile in a pregnancy after RYGB was characterized by frequent hypoglycemic events overnight. Daytime was characterized by glycemic variations with large amplitude, postprandial hyperglycemic spikes and hypoglycemic events [40].

A 2019 prospective observational study investigated cord blood glucose and insulin levels in a pregnancy after bariatric surgery (41 women), compared to levels in a pregnancy without maternal bariatric surgery (82 women). The control group was matched according to early pregnancy BMI. Investigators found no difference in cord blood glucose or insulin levels between both groups. Moreover, no association between maternal and neonatal insulin resistance was found [41]. This is in contrast to a 2009 report from Cleveland, (Ohio, USA) demonstrating an association between maternal obesity and fetal insulin resistance in normal pregnancy [45].

There is no evidence that treatment of GDM diagnosed by an OGTT in this population leads to improved pregnancy outcomes. This is highlighted by the study of Freitas et al. When applying the IADPSG criteria instead of the Carpenter and Coustan criteria in 30 post-RYGB pregnant women they, found a 50% increase in diagnosis of GDM, however, there was no difference in outcomes [38].

### 4.2. Is Abnormal Glucose Homeostasis a Main Culprit for Fetal Growth Retardation in A Pregnancy After Bariatric Surgery?

We identified 28 studies on the prevalence of SGA in a pregnancy after bariatric surgery (Table 3). These studies included smaller cohorts or case series, and larger population-based cohort studies. There were three nationwide studies from Denmark [46,47,48], three from Sweden [49,50,51] and four from the USA [52,53,54,55].

There are several findings that point to an association between SGA and abnormal glycemic levels after bariatric surgery. An Austrian paper investigated differences in frequently sampled 3 h 75 g OGTT and IVGTT in RYGB patients compared to obese and normal-weight control pregnant women. The results were correlated with pregnancy outcomes. A positive association was found between fetal growth and maternal glucose nadir during the OGTT [39]. A 2019 observational study investigated glucose levels during 75 g OGTT, as well as cord blood analyses, neonatal weight and body composition. In this report, a positive correlation was found between birthweight and post OGTT glucose level [41]. In an Israeli study, evaluating pregnancy outcomes after bariatric surgery between a group with hypoglycemic events compared to a group without hypoglycemic events, the hypoglycemic group presented with less GDM, but more SGA [43].

The timing of growth retardation in these fetuses is a topic of debate. A 2017 Austrian study found more pronounced hypoglycemia during an OGTT to be associated with reduced fetal abdominal circumference during the second trimester of pregnancy [62]. A 2020 prospective longitudinal study from the UK showed reduced fetal growth velocity starting in the third trimester, when compared to non-operated women with similar pre-pregnancy BMI [42]. A Danish national cohort study investigating 387 women after RYGB found an overall SGA proportion of 18.8%. In contrast to other studies, they found that early fetal growth was significantly impaired when compared to a historical cohort of 9450 singleton pregnancies in Denmark [46].

A large cohort study from Sweden with 670 pregnancies after bariatric surgery of which 98% were RYGB, noted more SGA when there was a longer surgery to conception interval [49]. On the other hand, a Turkish retrospective study investigating outcome after SG, found more SGA with a shorter surgery to conception interval [68]. A Danish national cohort study found no significant difference in SGA depending on a short or long surgery to conception interval (before or after 18 months: 19.7% versus 18%), there was a trend to lower SGA rates with increasing maternal BMI [46].

### 4.3. Prevalence of GDM in a Pregnancy After Bariatric Surgery

We identified 35 studies on the prevalence of GDM in a pregnancy after bariatric surgery (Table 4). These studies include smaller cohorts or case series, and larger population-based cohort studies. There are three population studies from Denmark [47,48,74], three from the USA [52,53,55], two from Sweden [49,50] and one from Australia [75]. Only 15 studies reported on the method of diagnosis of GDM: oral glucose tolerance test (OGTT), capillary blood glucose monitoring (CBGM) or measurement of glycosylated hemoglobin (HbA1c) (Table 4).

In most observational studies where a non-operated control group was used, bariatric surgery is associated with a lower prevalence of GDM (Table 4). Prevalence of GDM after bariatric surgery in these studies ranged from 0 to 12.9% [49,52,53,56,68,69,70,76,77,78,82,83]. Other studies comparing the GDM rate to a non-operated group showed a higher rate of GDM in women with a history of bariatric surgery [47,55,63,74,75]. There is significant heterogeneity in both case and control groups (Table 4).

The highest rate of GDM (26.34%) after bariatric surgery was found in a 2019 paper investigating the effect of gestational weight gain on pregnancy outcomes after bariatric surgery. A total of 337 pregnancies in women that underwent RYGB, SG and LAGB were studied. GDM diagnosis was based on patient charts, no information was available on the diagnostic method. Surprisingly, GDM was most prevalent in the group that had insufficient gestational weight gain (29.7%), whereas in the group of patients with excessive weight gain, a percentage of 22.1% was noted [65].

### 4.4. Impact of the Interval Between Bariatric Surgery And Pregnancy on the Prevalence of GDM

A recent Eurasian consensus paper recommends that pregnancy should be postponed until stable weight is achieved. In practice, this means 12 months after RYGB or SG [88]. The reason to delay a pregnancy is to prevent adverse outcome, mainly SGA. Several studies reported no difference in prevalence of GDM when comparing an interval shorter or longer than 1 year after bariatric surgery [48,60,68,80,84]. Other studies investigated longer intervals, of up to 2 years, and also showed no difference [49,57,71,81,87]. These effects were similar in different types of bariatric surgery including SG and RYGB.

There is however some evidence that a shorter interval between surgery and pregnancy is associated with a higher risk for hypoglycemia following a 100 g OGTT, as was shown by a study from Israel with patients after RYGB, SG and LAGB. In the hypoglycemia group, time from surgery to conception was significantly shorter (median 711 versus 1246 days, *p* = 0.002), risk of GDM tended to be lower (0% versus 10.9%, *p*-value: 0.3) and risk of SGA was higher (11.9% versus 1.7%, *p*-value: 0.3) [43]. A study investigating the effect of a 75 g OGTT in pregnant women with previous SG, compared women that became pregnant in the year after SG with women that conceived after twelve months. The early conception group reported more early (58%) and late (16%) dumping symptoms than the late conception group (14% and 9%, respectively) [68].

### 4.5. Impact of BMI After Bariatric Surgery on the Prevalence of GDM

In a case series of 102 post-bariatric-surgery pregnant women (type of surgery not reported), there was no difference in GDM prevalence between BMI ≥ 30 versus < 30 kg/m² [85]. A larger cohort study from Sweden confirmed this finding, showing that pre-pregnancy BMI and the amount of weight loss from bariatric surgery to early pregnancy does not modify the effect of bariatric surgery on the risk of developing GDM. In the bariatric surgery group with BMI < 42.1 versus ≥ 42.1 kg/m², GDM occurred in, respectively, 2.5% versus 1.5% of cases after bariatric surgery. In the bariatric surgery group with a decrease in BMI of ≥ 12.9 versus <12.9 kg/m², GDM occurred in 2.3% versus 1.6% of cases [49]. A French case series studied the effect of the amount of gestational weight gain in 337 pregnancies after bariatric surgery (RYGB, SG, LAGB), according to the 2009 Institute of Medicine (IOM) recommendations. Insufficient gestational weight gain (35%), as well as excessive gestational weight gain (38%) were frequent. The amount of gestational weight gain, however, had no statistically significant effect on the prevalence of GDM. GDM occurred in 29.7%, 28.1% and 22.1% of women with insufficient, adapted and excessive gestational weight gain, respectively (*p*-value: 0.36). There was no information on how GDM diagnosis was made. Insufficient gestational weight gain was positively correlated with low birth weight and SGA. Surgery to conception interval had no influence on the amount of gestational weight gain in this study [65].

### 4.6. Impact of Type of Bariatric Surgery on Prevalence of GDM

To our knowledge, there are no studies directly comparing the difference in GDM prevalence between SG and RYGB. Few studies have looked specifically at the prevalence of GDM after SG and report a percentage of 0–6.6% [57,68,71]. These studies used OGTT or CBGM. GDM prevalence in these studies was low when compared to studies in RYGB patients. A prospective observational study from the UK investigated pregnancy outcomes after restrictive versus malabsorptive bariatric surgery. The malabsorptive group consisted solely of women with RYGB; the restrictive group consisted of patients after SG or LAGB. GDM diagnosis was made with a 75 g OGTT. In the RYGB group, 0% of GDM was recorded in the SG-LAGB group, 21.1%. However, maternal post-prandial hypoglycemia was significantly more prevalent in the RYGB group (70%) compared with the SG-LAGB group (22%) [41]. The effect of SG on the development of postprandial hypoglycemia may be blunted by mixing LAGB with SG in one combined group, as a cohort study with 30 RYGB, 55 SG and 34 LAGB pregnant women showed postprandial hypoglycemia percentages of 83.3%, 54.5% and 11.8%, respectively [43].

## 5. Discussion

### 5.1. Summary of Findings

In this scoping review, we show that hypoglycemia as well as large and rapid glycemic excursions are underreported in pregnancies after bariatric surgery. These changes in glucose homeostasis may be responsible for adverse pregnancy outcomes such as SGA. The diagnosis of GDM in a pregnancy after bariatric surgery is challenging. Most studies reporting on GDM prevalence are based on an OGTT, although this test is considered unreliable.

### 5.2. Results in Relation to What We Already Know

Clinical practice recommendations on the diagnosis of GDM were published by the American Diabetes Association (ADA) in 2020 [89]. Screening for overt diabetes should be done at first prenatal contact using standard diagnostic criteria. In women without pre-existing diabetes mellitus, a test for GDM is advised at 24–28 weeks of gestation. GDM diagnosis can be made with one of two methods: a one-step 75 g OGTT (IADPSG criteria), or a two-step approach with a 50 glucose challenge test (GCT) followed by a 100 g OGTT in case of positive screening (Carpenter-Coustan criteria) [89]. A one-step 2 h 75 g OGTT is the gold standard test [90]. There is no evidence that treatment of GDM diagnosed by an OGTT in a population after bariatric surgery, leads to improved pregnancy outcomes. Since an OGTT is an unreliable and poorly tolerated test in women with a history of bariatric surgery, an alternative screening strategy for GDM is needed. Our research group recommended using CBGM daily before and after meals during 3–7 days at 24–28 weeks of pregnancy. Glycemic targets were based on American Diabetes Association (ADA) recommendations [91]. More research is needed to define optimal glycemic targets in this population. Each type of bariatric surgery has a specific glycemic footprint [17,20]. Both RYGB and SG are marked by postprandial hyperinsulinemic hypoglycemia, nightly hypoglycemia and wide glycemic variability [12,13]. This is influenced by specific anatomical alterations to the gastro-intestinal tract that alter glucose handling [19].

When examining SGA in a pregnancy after bariatric surgery, confounders such as smoking, lower socio-economic status and paternal BMI must be taken into account. Several studies note that patients after bariatric surgery are more likely to be smokers and have a lower socio-economic status [46,50]. A recent systematic review and meta-analysis showed that high paternal BMI can lead to distortion of fetal growth, leading to both SGA and LGA in normal pregnancies [92]. Levels of micronutrients, lipids, amino-acids and leptin have been shown to influence fetal growth. Micronutrient deficiencies are found to be more frequent after bariatric surgery, however, evidence linking these deficiencies to adverse pregnancy outcomes is weak [93]. The most common deficiencies after bariatric surgery are vitamin B12, vitamin D and other fat-soluble vitamins, folate, calcium, iron, proteins and fat [94]. Cord blood micronutrient levels in infants after RYGB showed deficiencies in calcium, zinc, iron and vitamin A when compared with neonates from lean, healthy mothers [63]. Maternal lipids and amino-acid levels have been linked to disturbed fetal growth [95]. Metabolomic and lipidomic research has shown a disturbed lipid profile in mothers and fetuses with intra-uterine growth restriction [96]. Low maternal leptin levels in mid pregnancy have been linked to SGA, a finding that persisted when adjusting for pre-pregnancy BMI [97].

In pregnancies after bariatric surgery, the impact of pre-pregnancy BMI on pregnancy outcomes is unclear. All three systematic reviews with meta-analysis report significant decrease in GDM prevalence after bariatric surgery. Subgroup analysis with matching for pre-pregnancy BMI no longer showed a significant improvement in GDM prevalence after bariatric surgery, concluding that the improvement in GDM is mainly weight-loss-driven [21,22]. On the other hand, other studies reported that pre-pregnancy BMI and the amount of weight loss from bariatric surgery to early pregnancy does not modify the effect of bariatric surgery on GDM improvement [49,85]. A recent prospective observational study from the UK compared maternal insulin resistance in pregnant women after RYGB versus pregnant women with similar BMI. A reduced insulin resistance, as assessed by HOMA-IR, was found in pregnancies after bariatric surgery [41]. In addition, a recent Austrian study found a significant reduction in HOMA-IR and liver fat in post-pregnancy NMR-spectroscopy in a RYGB group in comparison to an obese control group [40]. These results suggest that the positive effect of bariatric surgery on glucose homeostasis cannot solely be explained by weight reduction, but also by weight-independent improvement in insulin sensitivity [41]. Improved insulin sensitivity after SG and RYGB is present well before significant weight loss occurs [17,98]. Bariatric surgery has been proven to resolve or improve pre-existing type 2 diabetes mellitus during 3- to 5-year follow-up periods [99] and preoperative fasting insulin levels are shown to drop by 45% and 50% in the first 3 months after SG and RYGB, respectively [17]. These insights into the mechanisms of substantial insulin sensitivity improvement after bariatric surgery question the high rate of GDM in a pregnancy after bariatric surgery. On the other hand, we would expect insulin resistance during pregnancy to attenuate the severity of reactive hypoglycemia.

The reported dramatic changes in glucose homeostasis after bariatric surgery are variable depending on the timing after surgery [17]. In a pregnancy after bariatric surgery, the length of the interval does not seem to have an effect on the incidence of GDM. However, a shorter interval is related to more frequent occurrence of hypoglycemic events and symptoms consistent with dumping syndrome [43].

### 5.3. Novelty and Practical Implications

Most research groups divide bariatric surgery in malabsorptive versus restrictive types. SG and LAGB are therefore often considered as a similar operation. This is an archaic differentiation that does not take into account the current insights in the working mechanism of these complex metabolic surgeries [11]. For most bariatric surgery experts, LAGB is no longer considered as one of the gold standard bariatric interventions, since one out of three patients develops band erosions, and almost 50% of patients require band removal [10,100]. On the other hand, SG has proven to have metabolic effects that go well beyond the effect of restriction [101,102]. Since the efficacy of procedures improves, decision making on type of surgery is complex and is often still based on surgeons’ preference and experience. An ongoing prospective cohort study comparing the two major types of bariatric surgery will provide more accurate data [103].

Recent data suggest a correlation between hypoglycemia and reduced birthweight in a pregnancy after bariatric surgery [39,41,43]. Wide glycemic excursions and repeated periods of hypoglycemia could lead to the birth of an SGA baby. CGM could provide more detailed insight in glucose homeostasis during a pregnancy after bariatric surgery [36,40]. CGM should be blinded when used as a diagnostic tool, since misinterpretation can lead to aggravation of symptoms and glycemic excursions due to intake of foods with a high glycemic index [44]. Poor glycemic control is a known modifiable risk factor for GDM. In a pregnancy after bariatric surgery, optimizing glycemic time in range through targeted diet recommendations could further prevent adverse outcomes such as fetal growth impairment. Guidelines for adequate weight gain in a pregnancy after bariatric surgery should be reviewed [65].

### 5.4. Strengths and Limitations

We performed an extensive scoping review on the current knowledge of glucose homeostasis in a pregnancy after bariatric surgery. We specifically addressed the possible association with the development of SGA, as well as the positive impact on GDM prevalence. Because of the heterogeneity and scarcity of existing data, we did not perform a systematic review.

Research on glucose homeostasis after bariatric surgery consists of case-control and cohort studies. Larger, population-based studies have the disadvantage of using hospital or national register data, which makes accurate reporting of key concepts difficult. Most reports use a historical database, where often only ICD9 or ICD10 coding is available. Information on the type of bariatric surgery is not always available. SG and LAGB are often merged into one group of restrictive procedures. This is contrary to progresses made in the understanding of the working mechanism of these two very different operations [11]. In addition, different definitions of SGA were used across different studies. SGA is most commonly defined as a birthweight beneath the 10th percentile, however, some authors define SGA as below the 3rd percentile or less than two standard deviations below mean. The most commonly used diagnostic tool for GDM is a standard 2 h 75 g OGTT, however some studies used different amounts of glucose load. Different hypoglycemic cut-off values are used (50–60 mg/dL) as well as different timings of the glucose measurements. Furthermore, it is well established that an OGTT is unreliable to make a GDM diagnosis in this patient population [91].

### 5.5. Future Research

In the last 10 years, research has focused on the prevalence of improved and adverse pregnancy outcomes after bariatric surgery. There is now a growing interest and need to investigate the pathophysiology behind these outcomes. There is strong evidence that bariatric surgery induces a substantial shift in the intestinal microbiome [104]. In addition, these alterations have been linked to improved glucose homeostasis in both animal and human research [104]. Indeed, the intestinal microbiome has an important role in the pathophysiology of type 2 diabetes mellitus [105]. Evidence from metabolomics research and intestinal bacterial profiling in a pregnant population that underwent bariatric surgery is currently very limited. Very recently, a small study in pregnant women after bariatric surgery (25 with RYBG and eight with SG), showed that the subgroup with RYBG had significantly lower serum concentrations of branched-chain amino acids (leucine and isoleucine) and branched-chain fatty acids (isobutyrate) in the third trimester of pregnancy [106]. Furthermore, these changes were associated with a shift in the intestinal microbiome. Data from this research also suggest an association with reduced maternal insulin resistance, as well as the risk of delivery of an SGA infant [106]

Another mechanism might be through epigenetic changes. In 2013, a group from Canada and the US reported on differences in DNA methylation profile in offspring of women before versus after biliopancreatic diversion. Improved insulin sensitivity in offspring after biliopancreatic diversion was maintained through childhood [107].

## 6. Conclusions

Since an OGTT is unreliable to diagnose GDM in a pregnancy after bariatric surgery, the true incidence of GDM is unknown and future research is needed. Data from CBGM and CGM can give more accurate insights in glucose homeostasis in a pregnancy after bariatric surgery. More research is needed to develop accurate guidelines on gestational weight gain, ideal pre-pregnancy BMI, screening strategy and treatment of GDM in this specific population.

## Figures and Tables

**Table 1:** Maternal and neonatal outcomes in a pregnancy after bariatric surgery.

Study Characteristics	Cases/Controls *	Control	Outcome •					
			**LGA**	**GDM**	HDP	SGA	PB	PD
Galazis et al., UK, 2014 [21]	5.361/160.773	obese º or BMI matched	**0.46 †**	**0.47 †**	**0.45 ‡**	**1.93 †**	**1.31 ‡**	1.05~
Akhter et al., UK and Belgium, 2019 [8]	14.880/3.979.978	population	**0.42 †**	NR	NR	**2.13 †**	**1.35 †**	**1.38 ‡**
Kwong et al., Canada, 2018 [22]	8.364/2.780.717	population	0.31 °	0.21 °	0.38 °	2.18 °	1.33 °	ND
		pre-S		0.20 °		2.16 °		
		pre-P		1.04 °		2.23 °		
Yi et al., China, 2015 [23]	4.178/16.016	obese º	0.40 °	0.31 °	0.42 °	2.16 °	1.33 °	NR

BMI: body mass index, LGA: large for gestational age, GDM: gestational diabetes mellitus, HDP: hypertensive disorders of pregnancy, SGA: small for gestational age, PB: premature birth, PD: perinatal death, NR: not reported, ND: no difference * Cases represent number of pregnancies in women with a history of bariatric surgery/Controls are pregnancies in women without history of bariatric surgery, • odds ratios are reported for cases versus controls † *p*-value <0.001, ‡ *p*-value <0.05, ~no statistically significant difference, ° *p*-value not reported, º BMI ≥ 30 kg/m^2^, Pre-S: pre-surgery BMI matched, Pre-P: pre-pregnancy BMI matched.

**Table 2:** Glucose profile in a pregnancy after bariatric surgery.

Author, Year	Design	Cases *	Type of BS	Control Group	Test †	Hypoglycemia	Symptoms	Conclusion ‡
Andrade, 2016 [35]	Case series	38	NR	Not-pregnant post-BS	OGTT	5.26% (≤ 50 mg/dL)	26.31%	Lower risk of hypoglycemia during pregnancy versus non-pregnant post BS control.
Bonis, 2016 [36]	Case series	35	RYGB	no	CGM	NR	NR	High mean maximum IG, low mean minimum IG.
Feichtinger, 2017 [37]	Retrospective cohort	76	RYGB	BMI matched ∞	OGTT	54.8% (≤ 60 mg/dL)	NR	Lower fasting glucose, glycemic rise at 60 min, followed by hypoglycemia. Trend to positive association between FG and BW.
				lean ∞				
				obese ∞				
Freitas, 2014 [38]	Case series	30	RYGB	no	OGTT	25% (≤ 50 mg/dL)	57.9%	New diagnostic criteria for GDM increase diagnosis of GDM after RYGB with 50%, but no change in pregnancy outcome.
Göbl, 2017 [39]	Retrospective cohort	25	RYGB	morbidly obese ∞	OGTT (3 h)	90% (≤ 50 mg/dL)	NR	Positive association between FG and maternal glucose nadir level. IS during OGTT remained improved in RYGB versus BMI matched control.
				lean ∞	IVGTT			
Leutner, 2019 [40]	Prospective cohort	25	RYGB	obese ∞	OGTT	76% (< 54 mg/dL, ADA guidelines)	NR	High risk of nightly hypoglycemia.
					IVGTT			Postprandial hypoglycemia is GLP-1 regulated.
				lean ∞	CGM			
Maric, 2019 [41]	Prospective cohort	41	LAGB, SG,	pre-P ∞	OGTT HOMA-IR	43.90% (< 60 mg/dL)	NR	Lower HOMA-IR, birthweight and body fat, same cord HOMA-IR.
			RYGB					Positive association between postprandial glucose level and BW
Maric, 2020 [42]	Prospective cohort	47	20 SG and	pre-P ∞	OGTT	48.78% (< 60 mg/dL)	NR	Maternal glucose level at OGTT is positively associated with EFW and BW
			LAGB					
			27 RYGB					
Rottenstreich, 2018 [43]	Retrospective cohort	119	55 SG	no	OGTT (3 h, 100 gr)	49.6% (≤ 55 mg/dL)	NR	Hypoglycemia group: shorter surgery to conception interval, less GDM, more SGA.
			34 LAGB					
			30 RYGB					Hypoglycemia most prevalent after RYGB

BS: bariatric surgery, NR: not reported, OGTT: oral glucose tolerance test, RYGB: Roux-en-y gastric bypass, CGM: continuous glucose monitoring, IG: interstitial glucose, BMI: body mass index, FG: fetal growth, BW: birth weight, GDM: gestational diabetes mellitus, IVGTT: intravenous glucose tolerance test, IS: insulin sensitivity, ADA: American diabetes association, GLP-1: glucagon-like peptide 1, LAGB: laparoscopic adjustable gastric band, SG: sleeve gastrectomy, Pre-P: pre-pregnancy BMI matched, HOMA-IR: homeostasis model assessment of insulin resistance, EFW: estimated fetal weight, SGA: small for gestational age, * number of pregnant women with a history of bariatric surgery,∞ control group without history of bariatric surgery, † standard OGTT is 2 h 75 gr, non-standard method is described between brackets, percentage of pregnant women with a history of bariatric surgery displaying hypoglycemia and/or symptoms. Cut-off values for hypoglycemia are mentioned between brackets, ‡ main conclusion regarding pregnancies with maternal history of bariatric surgery.

**Table 3:** Prevalence of small-for-gestational-age infants (SGA) in a pregnancy after bariatric surgery.

Author, Year	Design	Cases *	Type of BS	Control Group ∞	SGA Definition •	SGA Prevalence
Adams, 2015 [52]	Cohort, population	764	RYGB	obese	≤ 10th percentile	OR: 2.16
Balestrin, 2019 [56]	Cohort, single center	93	Uncertain	obese	< 10th percentile	19.4% vs. 11.6%
Basbug, 2019 [57]	Case series, single center	23	SG	no	< 10th percentile	8.69%
Belogolovkin, 2012 [53]	Cohort, population	293	Uncertain	obese	< 10th percentile	2.69
Chevrot, 2016 [58]	Case control, single center	139	RYGB, SG, LAGB	pre-P	< 10th percentile	29% vs. 6% ~
				pre-S		17% vs. 9% ~
Costa, 2018 [59]	Case series, single center	39	RYGB, SG, LAGB	no	< 10th percentile	17.9%
Dolin, 2019 [60]	Cohort, single center	76	RYGB, SG, LAGB	no	< 10th percentile	0%
Ducarme, 2013 [61]	Cohort, multicenter	94	RYGB, LAGB	no	< 10th percentile	RYGB: 32.3%
						LAGB: 17.5%
Feichtinger, 2018 [62]	Case control, single center	43	RYGB	BMI matched	NR	26.2% vs. 4.7% ‡
Gascoin, 2017 [63]	Case control, single center	56	RYGB	lean	< 10th percentile	23% vs. 3.6%~
Gonzalez, 2015 [64]	Case series, multicenter	168	RYBG, SG, VBG, LAGB, BPD	no	< 3rd percentile	19.6%
Grandfils, 2019 [65]	Case series, multicenter	337	RYGB, SG, LAGB	no	< 10th percentile	25.81%
Hammeken, 2017 [66]	Cohort, single center	151	RYGB	pre-P	22% below average	2.67 OR
Hazart, 2017 [67]	Case series, single center	57	RYGB, LAGB, SG	no	< 10th percentile	36%
Johansson, 2015 [49]	Cohort, population	670	RYGB (98%), LAGB, other	pre-S	< 10th percentile	15.6% vs. 7.6% †, OR 2.20
Josefsson, 2011 [50]	Cohort, population	126	RYGB, VBG, LAGB	population	≥ 2 SD below mean	3.38% vs. 2.1% ~
Karadag, 2019 [68]	Cohort, single center	90	SG	obese	< 10th percentile	17.7% vs. 7.4%
Kjaer, feb 2013 [47]	Cohort, population	339	RYGB, LAGB	pre-P	≥ 2 SD below mean	OR: 2.29, RYGB: 2.78
Kjaer, mar 2013 [48]	Case series, population	286	RYGB	no	≥ 2 SD below mean	7.69%
Lesko, 2012 [69]	Cohort, single center	70	RYGB, LAGB	pre-P	NR	17.4% vs. 5.0% ~
				pre-S		(OR 3.94)
Norgaard, 2014 [46]	Cohort, population	387	RYGB	population	< 10th percentile	18.8%
Parent, 2017 [54]	Cohort, population	1859	RYGB, SG, LAGB, VBG	population	< 10th percentile	13.0% vs. 8.9% (RR 1.93 adjusted)
Parker, 2016 [55]	Cohort, population	1585	NR	obese population	< 10th percentile ••	5.7% vs. 2.2% †
Roos, 2013 [51]	Cohort, population	2562	RYGB, VBG, LAGB, other	BMI > 35	≥ 2SD below mean	5.2% vs. 3%†, OR 2
Rottenstreich, ma 2018 [70]	Case control, multicenter	119	SG	pre-S	< 10th percentile	14.3% vs. 4.2% ~
Rottenstreich, sep 2018 [71]	Case series, single center	154	SG	no	< 10th percentile	13.64%
Sancak, 2019 [72]	Case series, single center	44	SG	no	< 10th percentile	25%
Stentebjerg, 2017 [73]	Case series, single center	71	RYGB	no	≥ 2SD below mean	1.4%

BS: bariatric surgery, OR: odds ratio, RYGB: Roux-en-y gastric bypass, NR: not reported, LAGB: laparoscopic adjustable gastric band, VBG: vertical banded gastroplasty, SG: sleeve gastrectomy, Pre-P: pre-pregnancy BMI matched, Pre-S: pre-surgery BMI matched, BMI: body mass index, BPD: biliopancreatic diversion, SD: standard deviation, RR: relative risk, * number of pregnant women with a history of bariatric surgery, ∞ control group without history of bariatric surgery, † *p*-value < 0.001, ‡ *p*-value < 0.05, ~ no statistically significant difference, *p*-value not reported, • of mean birth weight, unless otherwise mentioned •• of mean weight during pregnancy.

**Table 4:** Prevalence of GDM in a pregnancy after bariatric surgery.

Author, Year	Design	Cases *	Type of BS	Control Group ∞	GDM Test •	GDM Prevalence
Adams, 2015 [52]	Cohort, population	764	RYGB	obese	NR	OR: 0.33 †
Amsalem, 2014 [76]	Retrospective cohort	109	LAGB, VBG	pregnancy before BS	NR	6.1% vs. 19% ~
Balestrin, 2019 [56]	Cohort, single center	93	Uncertain	obese	OGTT	12.9% vs. 26.5% ~
Basbug, 2019 [57]	Case series, single center	23	SG	no	OGTT	0%
Belogolovkin, 2012 [53]	Cohort, population	293	Uncertain	obese	NR	OR: 0.44
Berlac, 2014 [74]	Cohort, population	415	RYGB	pre-P	NR	9.2% vs. 8.1%
				lean		9.2% vs. 1.3% †
Burke, 2010 [77]	Retrospective cohort	354	87% RYGB	pregnancy before BS	NR	8% vs. 27%
						OR 0.23°
Chevrot, 2016 [58]	Case control, single center	139	RYGB, SG, LAGB	pre-P	NR	12 vs. 10% ~
				pre-S		12 vs. 23 % ~
Costa, 2018 [59]	Case series, single center	39	RYGB, SG, LAGB	no	NR	7.7%
De Alencar Costa, 2016 [78]	Retrospective case-control	63	RYGB	obese	NR	0% vs. 19.2% †
Dolin, 2019 [60]	Cohort, single center	76	RYGB, SG, LAGB	no	OGTT or CBGM	2.63%
Ducarme, 2013 [61]	Cohort, multicenter	94	RYGB, LAGB	no	OGTT (50 or 75 gr)	19.4%
Gascoin, 2017 [63]	Case control, single center	56	RYGB	lean	NR	1.78% vs. 0% ~
Gonzalez, 2015 [64]	Case series, multicenter	168	RYBG, SG, VBG, LAGB, BPD	no	OGTT (50 or 100 gr)	3%
Grandfils, 2019 [65]	Case series, multicenter	337	RYGB, SG, LAGB	no	NR	26.34%
Han, 2013 [79]	Case series	12	SG	no	NR	0%
Hazart, 2017 [67]	Case series, single center	57	RYGB, LAGB, SG	no	OGTT (50 gr)	18%
Ibiebele, 2019 [75]	Retrospective cross sectional	1484	RYGB, LAGB, SG	population	NR	10.8% vs. 8.3% †
Johansson, 2015 [49]	Cohort, population	670	RYGB (98%), LAGB, other	pre-S	OGTT or CBGM	1.9 vs. 6.8% †
						OR 0.25
Josefsson, 2011 [50]	Cohort, population	126	RYGB, VBG, LAGB	population	NR	no difference
Karadag, 2019 [68]	Cohort, single center	90	SG	obese	OGTT	6.6% vs. 29.6%
Kjaer, feb 2013 [47]	Cohort, population	339	RYGB, LAGB	pre-P	NR	8.9% vs. 7.1% ~
Kjaer, mar 2013 [48]	Case series, population	286	RYGB	no	NR	9.44%
Lesko, 2012 [69]	Cohort, single center	70	RYGB, LAGB	pre-P	OGTT (3 h)	0% vs. 9.3%, OR 0.04 ~
				pre-S		0% vs. 16.4%, OR 0.07 ~
Malakauskiene, 2019 [80]	Retrospective cohort	130	RYGB, LAGB	no	OGTT	2.31%
Parker, 2016 [55]	Cohort, population	1585	NR	obese	NR	7.3% vs. 4.4% ~
Rasteiro, 2018 [81]	Case series	86	RYGB, LAGB	no	OGTT or CBGM	19.77%
Rottenstreich, ma 2018 [70]	Case control, multicenter	119	SG	pre-S	NR	3.4% vs. 17.6% †
Rottenstreich, sep 2018 [71]	Case series, single center	154	SG	no	OGTT (100 gr, 3 h) or CBGM	2.6%
Rottenstreich, 2019 [82]	Retrospective case control	22	RYGB, LAGB, SG	pre-S	OGTT (100 gr, 3 h) or CBGM	9.1% vs. 36.4% †
Shai, 2014 [83]	Retrospective cohort	326	NR	obese	NR	10.1% vs. 14.7% ‡
Sheiner, 2011 [84]	Case series	489	RYGB, LAGB, VBG	no	NR	7.98%
Stone, 2011 [85]	Case series	102	NR	no	NR	11.76%
Watanabe, 2019 [86]	Case series, single center	24	RYGB, SG, LAGB, BPD-DS	no	HbA1c ≥ 6.5%	8.33%
Yau, 2017 [87]	Case series	49	RYGB, LAGB, SG	no	OGTT	2.44%

BS: bariatric surgery, GDM: gestational diabetes mellitus, RYGB: Roux-en-y gastric bypass, NR: not reported, vs: versus, OR: odds ratio, LAGB: laparoscopic adjustable gastric band, VBG: vertical banded gastroplasty, OGTT: oral glucose tolerance test, SG: sleeve gastrectomy, Pre-P: pre-pregnancy BMI matched, Pre-S: pre-surgery BMI matched, BMI: body mass index, BPD: biliopancreatic diversion, CBGM: capillary blood glucose monitoring, BPD-DS: biliopancreatic diversion with duodenal switch, HbA1c: glycosylated hemoglobin, * number of pregnant women with a history of bariatric surgery, ∞ control group without history of bariatric surgery, • standard OGTT is 2 h 75 gr, non-standard method is described between brackets, † *p*-value < 0.001, ‡ *p*-value < 0.05, ~ no statistically significant difference, *p*-value not reported.
